# 

**Published:** 1885-12

**Authors:** 


CONTENTS.

Vol. 32 No. 12

| | PAGE. | | PAGE. |
|---|---|---|---|
| New Arrangements | 1 | Drowning at Sea | 11 |
| Books and General Orders | 2 | To Make Yourself Unhappy | 12 |
| To Canvassers | 2 | Power of the Plug Hat | 12 |
| A Persian Workman | 4 | A New York Restaurant | 13 |
| Josh Billings | 5 | Prevention Better than Cure | 15 |
| Drooping Shoulders | 8 | Best Mode of Storing Ice | 17 |
| Babies Shoes | 8 | Sunlight in Stables | 17 |
| The Ancient Roman Circus | 9 | A Visit from Captain Kidd | 18 |
| Legal Phraseology | 9 | Wonders of the Sea | 19 |
| Vaccine Virus Victorious | 10 | | |

